# Novel chaotic oppositional fruit fly optimization algorithm for feature selection applied on COVID 19 patients’ health prediction

**DOI:** 10.1371/journal.pone.0275727

**Published:** 2022-10-10

**Authors:** Nebojsa Bacanin, Nebojsa Budimirovic, Venkatachalam K., Ivana Strumberger, Adel Fahad Alrasheedi, Mohamed Abouhawwash

**Affiliations:** 1 Faculty of Informatics and Computing, Singidunum University, Belgrade, Serbia; 2 Department of Applied Cybernetics,Faculty of Science, University of Hradec Kràlové, Hradec Kràalové, Czech Republic; 3 Department of Statistics and Operations Research, College of Science, King Saud University, Riyadh, Saudi Arabia; 4 Department of Mathematics, Faculty of Science, Mansoura University, Mansoura, Egypt; 5 Department of Computational Mathematics, Science and Engineering (CMSE), Michigan State University, East Lansing, MI, United States of America; Nottingham Trent University School of Science and Technology, UNITED KINGDOM

## Abstract

The fast-growing quantity of information hinders the process of machine learning, making it computationally costly and with substandard results. Feature selection is a pre-processing method for obtaining the optimal subset of features in a data set. Optimization algorithms struggle to decrease the dimensionality while retaining accuracy in high-dimensional data set. This article proposes a novel chaotic opposition fruit fly optimization algorithm, an improved variation of the original fruit fly algorithm, advanced and adapted for binary optimization problems. The proposed algorithm is tested on ten unconstrained benchmark functions and evaluated on twenty-one standard datasets taken from the Univesity of California, Irvine repository and Arizona State University. Further, the presented algorithm is assessed on a coronavirus disease dataset, as well. The proposed method is then compared with several well-known feature selection algorithms on the same datasets. The results prove that the presented algorithm predominantly outperform other algorithms in selecting the most relevant features by decreasing the number of utilized features and improving classification accuracy.

## 1 Introduction

Information and data are at the core of technological evolution. With the progress of technology, extensive datasets have improved the machine learning models in numerous domains but made the analysis of said datasets remarkably strenuous, considering that surplus, noisy and irrelevant data is abundant within the sets. That abundance of inconsequential data hinders the machine learning process, making it computationally expensive, frequently resulting in substandard performance and accuracy of the model.

Metaheuristic algorithms are exceptionally effective optimization methods, particulary in tackling demanding, high-dimensional issues. Imputable to their superb performance, researchers utilize metaheuristic algorithms to resolve feature selection problems. Eminent nature-inspired metaheuristic algorithms include evolutionary algorithms (EA), inspired by biological evolution (reproduction, mutation, recombination, and selection), and swarm intelligence (SI), which mimic the behavioural patterns of animals in a herd since they show substantial collective intelligence compared to the one of each individual.

### 1.1 Machine learning and feature selection

The focal objective of machine learning is successful output prediction of the algorithm for each input through experience [1]. Machine learning differentiates two types of scenarios: supervised and unsupervised. The one used in this manuscript—supervised learning [2], utilizes labelled datasets to train algorithms for accurate predictions of outcomes or data classification. A copious amount of data within datasets is what compels the machine learning model. Simultaneously, these large datasets, packed with redundant and inessential data, influence the machine learning process in regard to accuracy and computational complexity. Frequently, the said datasets are high-dimensional, which impedes the performance of the machine learning model, as well. This occurrence refers to the curse of dimensionality [3].

Hence, identifying essential information is crucial to tackling this issue. For this reason, the technique of dimensionality reduction [4], an action of reducing classification variables, is a main pre-processing task for machine learning. There are two approaches to dimension reduction: feature extraction and feature selection (FS). While feature extraction [5] generates new variables derived from the primary set of data, FS selects a subset of relevant informative variables for desired objective.

The purpose of FS is to determine the relevant subset from high-dimensional data sets eliminating the insignificant features, thus enhancing the classification accuracy for machine learning. There are three feature selection methods: filter, wrapper and the embedded methods, as per G. Chandrashekar et al. [6].

Wrapper methods utilize learning algorithms, like classifiers, to evaluate feature subsets to find relevant features. Wrapper methods yield the best-performing feature subsets (smaller subsets and higher classification accuracy) but are computationally demanding.

Filter methods do not use a training process and instead designate a score to feature subsets using a measure. Due to that, this method is not as computationally demanding as the wrapper and creates a universal set (unadjusted to a specific prediction model).

The embedded method employs FS as a segment of the model-creating process, i.e. methods perform FS during the model training. These methods are more accurate than filters, with the same execution speed. With computational complexity in mind, embedded methods are in between the methods mentioned above.

### 1.2 Paper goal and structure

One might wonder whether a new optimization method is needed, considering that there are numerous optimization algorithms in studies that carry out the task rather well.

The No Free Lunch (NFL) theorem [7] demonstrates that none of the algorithms can resolve all optimization issues. Meaning, present-day algorithms for feature selection are not capable of solving all feature selection issues. This inspires researchers to enhance and adapt existing algorithms or present new algorithms, to cope with a wide range of problems.

Optimization algorithms are coping with providing optimal informative subsets within high-dimensional data sets. Therefore, a new metaheuristic algorithm is demanded to enhance resolving feature selection problems.

This manuscript proposes a well-known swarm intelligence metaheuristic, fruit fly optimization algorithm (FFO), improved and adapted for solving FS problems in a wrapper-based approach. The goal of the presented research in this paper is to enhance solving feature selection problems with a proposed chaotic oppositional fruit fly optimization (COFFO) algorithm by obtaining high classification accuracy on different datasets. COFFO is tested on ten unconstrained benchmark functions (CEC2019), then on 21 standard datasets taken from the Univesity of California, Irvine (UCI) repository and Arizona State University (ASU), along with the coronavirus disease (COVID-19) dataset. Additionally, the proposed method is compared with several well-known feature selection algorithms on the same datasets. The results prove that the presented COFFO predominantly outperform other algorithms in selecting the most relevant features.

This following research questions inspire this work:

- Is it achievable to further improve the original FFO algorithm for high-dimensional feature selection problems?
- Is it attainable to further improve the solving of FS problems with the proposed COFFO by enhancing the accuracy and selecting features with a higher impact on the target variable?

The contributions of this research are summarized as follows:

- The proposal of COFFO, an upgraded variant of the original FFO, is suitable for solving even high-dimensional FS problems.
- This robust method is implementing chaotic behaviour and opposition-based learning to improve population diversity and exploratory capacity of FFO.
- After extensive testing of the proposed method and comparing it with other well-known feature selection algorithms, the conclusion is that solving of FS problems is furthermore improved.
- Implementing FFO and COFFO algorithms in COVID-19 patient health prediction is a beneficial contribution to medicine.

The structuring of this paper is as follows. Section 2 provides a brief overview of swarm intelligence algorithms and their applications in various fields.Section 3 presents the basic fruit fly optimization algorithm, summarizes its downsides before proposing an improved variation of this promising algorithm. Sections 4 and 5 present results of the presented aproach, as well as a comparison with other well-known methods for standard CEC2019 benchmarks and then for twenty-one standard datasets taken from the UCI and ASU. Section 6 displays the application of COFFO on COVID-19 datasets. Section 7 discusses advantages and disadvantages of COFFO. Lastly, Section 8 draws conclusions and future directions.

## 2 Related works

Nature-inspired metaheuristic algorithms have shown high efficiency in solving numerous optimization problems and, as such, are in the lead as of recent apropos solving complex real-world problems. Metaheuristic algorithms enable attaining suboptimal solutions in a reasonable time frame.

The literature proposes numerous methods to mimic the behavioural patterns of animals in a herd since they show substantial collective intelligence compared to the one of each individual. Ant colony optimization (ACO) [8], the whale optimization algorithm (WOA) [9], grey wolf optimizer algorithm (GWOA) [10], artificial bee colony (ABC) [11], grasshopper optimization algorithm (GOA) [12], particle swarm optimization (PSO) [13] and salp swarm algorithm (SSA) [14] are among the most popular ones.

Various problems in diverse disciplines have benefited from SI problem-solving solutions, such as medical applications for diagnosing serious diseases in early stages [15] or the COVID-19 cases predictions [16], problems with optimization of artificial neural network parameters [17–20], the management and normal functioning of wireless sensor networks [21–23] up to resolving issues in cloud computing [24–26]. The paper [27] offered an extensive analysis of metaheuristics for the feature selection problem.

Apropos COVID-19 patient diagnostic, paper [28] proposed a hybrid FS method to find optimal subset of features obtained from the chest computed tomography images. Research [29] introduced a deep network model to pinpoint the COVID-19 disease built on X-ray images. Relief-based FS algorithm suggested in [30], is used to filter the unnecessary features in COVID-19 prediction.

Multiple swarm intelligence algorithms are employed to solve the feature selection problem [31–33]. For that purpose, copious binary metaheuristic methods are created, predominantly for wrapper-based FS. The two essential terms, transfer function (TF) and binarization, are utilized. Binary particle swarm optimization (BPSO) is presented in [34]to resolve discrete problems. Dragonfly algorithm (DA) [35], created to solve continuous optimization problems by simulating the swarming patterns of a dragonfly, got its binary version BDA [36], which utilizes transfer functions that differ in time. Heavy exploitation of BDA can produce a local optima problem, thus failing to obtain the global optimal solution. An improved version, the hyper learning binary dragonfly algorithm (HLBDA) [37], uses the hyper learning strategy enabling the dragonfly to learn from both personal and global best solutions throughout the search phase. Research [38] presented a binary artificial bee colony (BABC) established on the Jaccard coefficient dissimilarity, but the method has a complex structure. A binary version of a grasshopper optimization algorithm (BGOA) [39] employs sigmoid and V-shaped transfer functions and has an integrated mutation operator to improve the diversification stage.

## 3 Proposed method

First, the original fruit fly optimization algorithm is introduced, followed by the proposed hybrid method for feature selection problem.

### 3.1 Original fruit fly optimization algorithm

Fruit fly optimization algorithm, proposed by Prof. Pan [40, 41], is a somewhat new nature-inspired optimization algorithm. In contrast to other metaheuristic algorithms, FFO is easy to comprehend and apply, thanks to the simple computational operation.

This method is an auspicious swarm intelligence algorithm motivated by the knowledge of the foraging behavioural patterns of fruit flies. The fruit fly surpasses other species relating to vision and olfaction, on which they predominantly rely—fruit flies can gather miscellaneous aerial smells, despite the source of food being far away. Throughout the scouring activity, fruit flies scout and locate food sources surrounding the swarm and estimate the smell concentration for each food source. When the best location with the highest smell concentration is detected, the swarm navigates towards it.

Undeniably, the process of effective communication and teamwork among individual fruit flies is essential to accomplishment in the tactics of solving an optimization problem. The algorithm contains four phases:

- initialization,
- osphresis foraging,
- population evaluation,
- vision.

Initially, the parameters are set—the maximum number of iterations and population size. The solutions, i.e. fruit flies, are initiated randomly (1)

$$\begin{array}{c} X_{i,j}=rand\left(UB_{j}-LB_{j}\right)+LB_{j}, \end{array}$$ (1)

where *X*_*i*,*j*_ implies *i*-th solution and *j* denotes the element’s position in the *i*-th solution. *LB* represents lower bound, while *UB* represents an upper bound, and *rand* is a random number from the uniform distribution.

Then, the position update of each solution occurs in accordance with the osphresis foraging phase. The solutions are distributed randomly from the current location, formulated in (2)

$$\begin{array}{c} X_{i,j}^{\left(t+1\right)}=X_{i,j}^{\left(t\right)}\pm rand\left(\right), \end{array}$$ (2)

where $X_{i,j}^{\left(t+1\right)}$ represents the new position, $X_{i,j}^{\left(t\right)}$ represent current solution, *rand*() ∈[−1, 1], while *t* denotes the iteration counter. Following the position update, distance and smell are calculated. Then, the computation of smell concentration—the function of smell (fitness function), for each solution, ensues. If the solution’s new best fitness function value is better than the previous best, then the solution’s new location with the best fitness function value will replace all solution’s positions. Otherwise, the old solution’s location will remain. This process represents the vision foraging phase of the algorithm. The algorithm continues until satisfying the stopping criteria and yields the best solution.

### 3.2 Motivation for improvement and proposed chaotic oppositional fruit fly optimization algorithm

The adaptation of a FFO algorithm to a particular problem is uncomplicated since its somewhat simple configuration. Notwithstanding the good performance of basic FFO [40, 41], by performing extensive practical simulations on a wide range of benchmark instances from Congress on Evolutionary Computation (CEC), it was observed that the basic FFO can be further improved.

Namely, basic FFO in some runs, due to stochastic nature, exhibits not so good exploration ability, because it performs fixed position update strategy and can be easily stuck in the local optima. Moreover, it was suggested that its exploitation capabilities can be further enhanced.

Method proposed in this study addresses above mentioned drawbacks by implementing opposition-based learning (OBL) and chaotic behavior in the original FFO approach. Inspired by the proposed modifications, method showed in this study is named chaotic oppositional fruit fly optimization (COFFO) algorithm.

The OBL was introduced for the first time in 2005 by Tizhoosh [42] and it was proved that this mechanism can substantially improve exploration and exploitation abilities of metaheuristics method [42, 43].

The OBL mechanism is mathematically described as follows: let *x*_*j*_ denotes *j*-the parameter of solution *x* and the $x_{j}^{o}$ represents its opposite number. The opposite number of *j*-th parameter of individual *x* calculates as follows:

$$\begin{array}{c} x_{j}^{o}=LB_{j}+UB_{j}-x_{j}, \end{array}$$ (3)

where *x*_*j*_ ∈ [*LB*_*j*_, *UB*_*j*_] and *LB*_*j*_, *UB*_*j*_ ∈ *R*, ∀*j* ∈ 1, 2, 3, …*D*. Parameter *D* represent the number of solution dimensions (parameters).

In complex implementations, the imbalance between exploitation and exploration and the randomness of the initialization phase causes the entrapment of optimization algorithms in the local optima. Literary manuscripts propose chaos theory as one of the methods for resolving this issue. Chaos optimization algorithm (COA) [44] is an example of chaos implementation that exploits the nature of the chaotic structure. Classification performance can be improved by applying chaotic system rather than the random parameter values [45]. Examples of these implementations are the following: chaotic whale optimization algorithm (CWOA) [46], chaotic grey wolf optimization (CGWO) [47] and chaotic grasshopper optimization algorithm (CGOA) [48].

Chaos represents a non-linear occurrence of a dynamic but deterministic system with stochastic patterns that is exceedingly receptive to its initial conditions. Although multiple chaotic maps exist, experimental testing shows that the logistic map provided the best results with the introduced COFFO. Chaotic-based search strategy implementation in the presented COFFO is generated by the chaotic sequence in line with the limitations of a specific problem. When the sequence is created, individuals employ it to explore the search space. The COFFO uses chaotic sequence *β*, which starts from arbitrary initial number *β*_0_ created by the logistic mapping. Logistic map executes in *K* steps in a following way:

$$\begin{array}{c} \beta_{i,j}^{k+1}=\mu \beta_{i,j}^{k}\left(1-\beta_{i,j}\right),\;\;k=1,2,...K, \end{array}$$ (4)

where $\beta_{i,j}^{k}$ and $\beta_{i,j}^{k+1}$ denote chaotic variable for *j*-th component of the *i*-th solution in steps *k* and *k*+ 1, respectively, while *μ* denotes chaotic control parameter. The *μ* typically has the value 4 [49], a value used in this work as well, to guarantee chaotic behaviour of individuals, the *β*_*i*,*j*_ ≠ 0.25, 0.5 and 0.75 and *σ*_*i*, *j*_ ∈ (0, 1).

Action of mapping solutions onto generated chaotic sequences is achieved with following formulation for each component *j* of individual *i*:

$$\begin{array}{c} X_{i}^{c}=\beta_{i}X_{i}, \end{array}$$ (5)

where $X_{i}^{c}$ is the new location of individual *i* after chaotic disruptions.

To establish an initial population of high quality, proposed COFFO first incorporates chaotic-opposition-based initialization, which is shown in Algorithm 1.

**Algorithm 1** Chaotic-opposition-based initialization pseudo-code

Step 1: Generate standard random population *P* of *N* solutions with expression: *X*_*i*_ = *LB*+ (*UB*−*LB*) ⋅ *rand*(0, 1), *i* = 1, …*N*, where *rand*(0, 1) denotes pseudo-random number from the interval [0, 1].

Step 2: Generate opposition population *P*^*o*^ for first *N*/2 individuals by triggering OBL using Eq (3)

Step 3: Generate chaotic population *P*^*c*^ of *N*/2 individuals by mapping solutions from *P* to chaotic sequences using expressions (4) and (5).

Step 4: Calculate fitness of all solutions from *P*, *P*^*o*^ and *P*^*c*^.

Step 5: Sort all individuals from *P* ∪ *P*^*o*^ ∪ *P*^*c*^ according to fitness.

Step 6: Select *N* best individuals from sorted set *P* ∪ *P*^*o*^ ∪ *P*^*c*^ for initial population.

In this way, initial population *P* is closer to optimum region of the search space and the COFFO can utilize more iterations for performing exploitation and exploration in this region.

However, despite of novel initialization strategy, exploitation ability in later cycles should also be improved and for this reason, COFFO incorporates chaotic local search (CLS) strategy which is executed around the current global best (X*) solution. Throughout every step *k*, new X*, represented as *X*^′*^, is created by applying Eqs (6) and (7), for each component *j* of X*:

$$\begin{array}{c} X_{j}^{'*}=\left(1-\lambda \right)x_{j}^{*}+\lambda S_{j} \end{array}$$ (6)

$$\begin{array}{c} S_{j}=l_{j}+\beta_{j}^{k}\left(u_{j}-l_{j}\right), \end{array}$$ (7)

where $\beta_{j}^{k}$ is calculated by Eq (4), while λ is a dynamic shrinkage parameter, depending on the maximum number of fitness function evaluations (*maxFFE*) and the current fitness function evaluation (*FFE*) in the algorithm’s execution:

$$\begin{array}{c} \lambda =\frac{maxFFE-FFE+1}{maxFFE} \end{array}$$ (8)

The use of dynamic λ allows for a better exploitation-exploration equilibria to be built around the X*. Earlier stages of execution explore a larger search area around the X*, while later stages emphasize on fine-tuned exploitation. Alternatively, the *maxFFE* and *FFE* can be replaced with *T* and *t* when the maximum number of iterations is considered as the termination condition.

In that manner, utilization of the CLS strategy is an attempt to enhance X* in *K* steps. If the *X*^′*^ achieves better fitness value than the X*, then the CLS procedure terminates and the *X*^′*^ replaces X*. Nevertheless, if X* cannot improve in *K* steps, it remains in the population.

Again, by conducting empirical experiments with CLS, it was observed that this mechanism should not be triggered too early. If it is executed in early iterations, when the search process did not converge enough, many *FFEs* are wasted. For that reason, additional control parameters, CLS trigger (*clst*) is incorporated that determines whether or not the CLS around X* will be executed. The value of this parameter is determined empirically, as it is shown in Section 4.

Taking all into consideration, workings of proposed COFFO are summarized in Algorithm 2.

**Algorithm 2** Proposed COFFO pseudo-code

Generate initial population according to Algorithm 1

Set the *FFEs* to 0 and define the termination criteria (*maxFFEs*)

Evaluate the fitness of each individuals

**while** *FFEs* < *maxFFEs* **do**

 **for** *i* = 1 to *N* **do**

  Update the position according to FFO updating mechanism by Eq (2)

 **end for**

 Determine the X* solution

 **if** *FFEs* > *clst* **then**

  Perform CLS strategy by using Eqs 6 and 7

  Adjust λ by applying expression 8

 **end if**

**end while**

Return the X* solution

Complexity in metaheuristics is measured by the number of *FFEs*, as the *FFE* is the most demanding operation. For the suggested algorithm, it can be calculated in a following way:

$$O\left(COFFO\right)=O\left(N\cdot 2\right)+O\left(N\cdot T\right)+O\left(T\right),$$

where *N* is the number of solutions in a population, *T* is the maximum number of iterations. This equation stands in a worst-case scenario, i.e. in each iteration, a chaotic local search is executed, and one solution evaluated. *maxFFE* is used as a termination condition in simulations for unbiased comparative analysis, even if the proposed algorithm uses more *FFEs* than some algorithms in each iteration.

## 4 Simulation and comparative analysis for unconstrained functions

First, the presented approach is substantiated on unconstrained benchmark functions.

Ten CEC2019 functions [50] are utilized to validate the performance of the presented method, before applying it to a real-world task. The original FFO and nine other metaheuristic-based algorithms: elephant herding optimization (EHO) [51], EHO improved (EHOI) [52], sine cosine algorithm (SCA) [53], salp swarm algorithm (SSA) [14], grasshopper optimization algorithm (GOA) [12], moth-flame optimization (MFO) [54], particle swarm optimization (PSO) [13], whale optimization algorithm (WOA) [9], biogeography-based optimization (BBO) [55] are tested on ten recent benchmark function set, presented on the Congress on Evolutionary Computation 2019 (CEC2019) [50], under similar circumstances. Additionally, the existing PSO embedded with chaotic opposition-based initialization (COPSO) is added for a more comprehensive comparative analysis. These results are then compared to the results gained by the presented algorithm.

The CEC2019 bound-constrained benchmark function characteristics are given in Table 1

**Table 1:** CEC 2019 benchmark characteristics.

Function	*F*_*i*_ = *F*_*i*_(x*)	D	Search Range
CEC1	1	9	[-8192, 8192]
CEC2	1	16	[-16384, 16384]
CEC3	1	18	[-4,4]
CEC4	1	10	[-100,100]
CEC5	1	10	[-100,100]
CEC6	1	10	[-100,100]
CEC7	1	10	[-100,100]
CEC8	1	10	[-100,100]
CEC9	1	10	[-100,100]
CEC10	1	10	[-100,100]

Research paper [52] provides the simulation results of previously mentioned algorithms for the same benchmarks. The same experiments are conducted anew to corroborate results from [52] and from an unbiased comparative analysis. Control parameters used to test methods in [52], population size *N* = 50 and a maximum number of iterations *maxIter* = 500, might prompt a very biased comparative analysis considering not all algorithms use the same number of fitness function evaluations (*FFEs*) in one iteration. In the initialization phase, most of these algorithms use *N* evaluations and then, in every iteration for each individual in the population, execute one more *FFE*. Hence, the termination condition *maxFFE* = *N* + N*maxIter is set to 25, 050 for all methods. That way, the same experimental conditions are established as in the [52], and the comparative analysis is unbiased.

Other parameters are set as follows: the size of the population is fixed at *N* = 50 and the *clst* expression was empirically determined as *maxFFEs*/3, which is in this case 8,350. This experiment is redone in 30 independent runs. Table 2 shows the control parameters for COFFO used throughout the unconstrained benchmark function experiment.

**Table 2:** COFFO control parameters.

Parameter Description	Notation	Value
Size of population	*N*	50
Termination condition in terms of *FFEs*	*maxFFEs*	25,050
Chaotic parameter	*K*	4
CLS trigger	*clst*	8,350

Control parameters for metaheuristics, used in this comparative analysis, were set as suggested in the original manuscripts.

Table 3 displays the gained experimental results—corresponding mean values and standard deviations of the presented and comparable methods. The best mean value is displayed in bold style for every benchmark instance, while the best standard deviation value is in italic, for easier reading. The obtained results of EHO, EHOI, SCA, SSA, GOA, MFO, PSO, WOA and BBO are slightly different from the results in the paper [52] due to the stochastic nature of observed algorithms.

**Table 3:** Mean fitness and standard deviation results of compared approaches on CEC2019 benchmark functions.

Function	Stats	EHOI	EHO	SCA	SSA	GOA	WOA	BBO	MFO	PSO	COPSO	FFO	COFFO
CEC1	mean	4.76 ⋅ 10^4^	1.35 ⋅ 10^7^	9.83 ⋅ 10^9^	3.21 ⋅ 10^9^	1.61 ⋅ 10^10^	1.03 ⋅ 10^10^	3.52 ⋅ 10^10^	7.17 ⋅ 10^9^	6.75 ⋅ 10^11^	4.17 ⋅ 10^10^	1.46 ⋅ 10^3^	**1.19** **⋅** **10**^**2**^
	std	2.69 ⋅ 10^3^	7.74 ⋅ 10^6^	5.47 ⋅ 10^8^	2.07 ⋅ 10^9^	1.14 ⋅ 10^10^	8.81 ⋅ 10^9^	2.55 ⋅ 10^10^	7.58 ⋅ 10^9^	6.53 ⋅ 10^11^	4.76 ⋅ 10^10^	4.46 ⋅ 10^2^	*1.32 ⋅ 10* ^*1*^
CEC2	mean	1.70 ⋅ 10^1^	1.72 ⋅ 10^1^	1.75 ⋅ 10^1^	1.73 ⋅ 10^1^	1.74 ⋅ 10^1^	1.73 ⋅ 10^1^	8.87 ⋅ 10^1^	1.74 ⋅ 10^1^	8.56 ⋅ 10^1^	1.64 ⋅ 10^2^	2.79 ⋅ 10^0^	**2.43** **⋅** **10**^**0**^
	std	*1.07 ⋅ 10* ^*−15*^	4.82 ⋅ 10^−15^	3.98 ⋅ 10^−2^	8.07 ⋅ 10^−5^	1.40 ⋅ 10^−2^	2.77 ⋅ 0^−3^	2.49 ⋅ 10^1^	3.83 ⋅ 10^−15^	3.96 ⋅ 10^1^	1.27 ⋅ 10^1^	1.38 ⋅ 10^2^	8.14 ⋅ 10^1^
CEC3	mean	1.27 ⋅ 10^1^	1.27 ⋅ 10^1^	1.27 ⋅ 10^1^	1.27 ⋅ 10^1^	1.27 ⋅ 10^1^	1.27 ⋅ 10^1^	1.27 ⋅ 10^1^	1.27 ⋅ 10^1^	1.27 ⋅ 10^1^	1.27 ⋅ 10^1^	9.71 ⋅ 10^0^	**5.29** **⋅** **10**^**0**^
	std	*1.90 ⋅ 10* ^*−15*^	*1.90 ⋅ 10* ^*−15*^	1.09 ⋅ 10^−4^	2.33 ⋅ 10^−15^	1.21 ⋅ 10^−4^	1.39 ⋅ 10^−7^	2.58 ⋅ 10^−7^	3.39 ⋅ 10^−5^	6.61 ⋅ 10^−4^	3.95 ⋅ 10^−4^	4.27 ⋅ 10^−1^	2.64 ⋅ 10^−1^
CEC4	mean	1.28 ⋅ 10^1^	1.55 ⋅ 10^1^	8.32 ⋅ 10^2^	3.25 ⋅ 10^1^	1.51 ⋅ 10^2^	2.65 ⋅ 10^2^	6.95 ⋅ 10^1^	1.38 ⋅ 10^2^	6.92 ⋅ 10^1^	2.83 ⋅ 10^1^	3.99 ⋅ 10^0^	**1.00** **⋅** **10**^**0**^
	std	3.84 ⋅ 10^0^	6.37 ⋅ 10^0^	2.91 ⋅ 10^2^	1.32 ⋅ 10^1^	1.49 ⋅ 10^2^	1.28 ⋅ 10^2^	2.35 ⋅ 10^1^	1.57 ⋅ 10^2^	8.02 ⋅ 10^0^	7.43 ⋅ 10^0^	*4.21E ⋅ 10* ^*−1*^	5.16 ⋅ 10^−1^
CEC5	mean	1.05 ⋅ 10^0^	1.07 ⋅ 10^0^	2.23 ⋅ 10^0^	1.35 ⋅ 10^0^	1.33 ⋅ 10^0^	1.67 ⋅ 10^0^	1.31 ⋅ 10^0^	1.13 ⋅ 10^0^	1.55 ⋅ 10^0^	1.33 ⋅ 10^0^	1.02 ⋅ 10^0^	**1.00** **⋅** **10**^**0**^
	std	2.12 ⋅ 10^−2^	2.20 ⋅ 10^−2^	7.79 ⋅ 10^−2^	1.12 ⋅ 10^−1^	1.41 ⋅ 10^−1^	4.18 ⋅ 10^−1^	9.68 ⋅ 10^−2^	8.23 ⋅ 10^−2^	1.16 ⋅ 10^−1^	1.37 ⋅ 10^−1^	2.34 ⋅ 10^−2^	*2.10 ⋅ 10* ^*−2*^
CEC6	mean	8.334 ⋅ 10^0^	9.45 ⋅ 10^0^	1.04 ⋅ 10^1^	3.79 ⋅ 10^0^	6.19 ⋅ 10^0^	9.14 ⋅ 10^0^	5.78 ⋅ 10^0^	4.92 ⋅ 10^0^	1.03 ⋅ 10^1^	6.56 ⋅ 10^0^	**1.61** **⋅** **10**^**0**^	1.87 ⋅ 10^0^
	std	8.56 ⋅ 10^−1^	1.24 ⋅ 10^0^	7.58 ⋅ 10^−1^	1.27 ⋅ 10^0^	1.37 ⋅ 10^0^	1.04 ⋅ 10^0^	6.43 ⋅ 10^−1^	2.21 ⋅ 10^0^	6.78 ⋅ 10^−1^	7.13 ⋅ 10^−1^	6.17 ⋅ 10^−2^	*5.41 ⋅ 10* ^*−2*^
CEC7	mean	1.42 ⋅ 10^2^	1.81 ⋅ 10^2^	6.38 ⋅ 10^2^	2.89 ⋅ 10^2^	2.87 ⋅ 10^2^	4.53 ⋅ 10^2^	4.92 ⋅ 10^0^	3.19 ⋅ 10^2^	6.97 ⋅ 10^2^	3.93 ⋅ 10^2^	5.91 ⋅ 10^0^	**2.33** **⋅** **10**^**0**^
	std	3.97 ⋅ 10^2^	1.43 ⋅ 10^2^	1.38 ⋅ 10^2^	2.35 ⋅ 10^2^	1.74 ⋅ 10^2^	2.17 ⋅ 10^2^	1.26 ⋅ 10^2^	2.10 ⋅ 10^2^	1.62 ⋅ 10^2^	1.36 ⋅ 10^2^	1.07 ⋅ 10^2^	*4.14 ⋅ 10* ^*1*^
CEC8	mean	2.69 ⋅ 10^0^	3.15 ⋅ 10^0^	5.77 ⋅ 10^0^	5.08 ⋅ 10^0^	5.49 ⋅ 10^0^	5.75 ⋅ 10^0^	4.81 ⋅ 10^0^	5.45 ⋅ 10^0^	5.10 ⋅ 10^0^	5.22 ⋅ 10^0^	1.15 ⋅ 10^0^	**1.02** **⋅** **10**^**0**^
	std	8.63 ⋅ 10^−1^	1.17 ⋅ 10^0^	*5.50 ⋅ 10* ^*−1*^	6.42 ⋅ 10^−1^	8.13 ⋅ 10^−1^	7.76 ⋅ 10^−1^	1.13 ⋅ 10^0^	5.78 ⋅ 10^−1^	7.38 ⋅ 10^−1^	7.56 ⋅ 10^−1^	7.99 ⋅ 10^−1^	5.64 ⋅ 10^−1^
CEC9	mean	2.29 ⋅ 10^0^	2.41 ⋅ 10^0^	9.75 ⋅ 10^1^	2.38 ⋅ 10^0^	2.45 ⋅ 10^0^	5.16 ⋅ 10^0^	3.75 ⋅ 10^0^	2.46 ⋅ 10^0^	2.65 ⋅ 10^0^	2.54 ⋅ 10^0^	3.91 ⋅ 10^0^	**2.15** **⋅** **10**^**0**^
	std	*6.34 ⋅ 10* ^*−3*^	1.38 ⋅ 10^−2^	9.23 ⋅ 10^1^	4.52 ⋅ 10^−2^	7.28 ⋅ 10^−2^	7.59 ⋅ 10^−1^	2.51 ⋅ 10^−1^	6.24 ⋅ 10^−2^	9.41 ⋅ 10^−2^	5.84 ⋅ 10^−2^	2.85 ⋅ 10^−1^	6.38 ⋅ 10^−3^
CEC10	mean	**1.92** **⋅** **10**^**1**^	2.11 ⋅ 10^1^	2.08 ⋅ 10^1^	2.03 ⋅ 10^1^	2.00 ⋅ 10^1^	2.05 ⋅ 10^1^	2.07 ⋅ 10^1^	2.02 ⋅ 10^1^	2.06 ⋅ 10^1^	2.06 ⋅ 10^1^	2.10 ⋅ 10^1^	1.95 ⋅ 10^1^
	std	1.52 ⋅ 10^0^	1.03 ⋅ 10^−1^	8.27 ⋅ 10^−2^	8.29 ⋅ 10^−2^	9.24 ⋅ 10^−2^	4.91 ⋅ 10^−2^	2.29 ⋅ 10^−2^	1.48 ⋅ 10^−1^	1.07 ⋅ 10^−1^	8.31 ⋅ 10^−2^	*1.14 ⋅ 10* ^*−5*^	9.09 ⋅ 10^−5^

From the results in Table 3, it is apparent that the presented method outperformed other tested algorithms. COFFO has the best mean value regarding eight functions (CEC1, CEC2, CEC3, CEC4, CEC5, CEC7, CEC8 and CEC9). The original FFO achieved the best mean value on CEC6 test instance, followed by COFFO. EHOI performed best on function CEC10, marginally in front of COFFO. COPSO obtained a better mean fitness value than the original PSO on seven functions due to chaotic opposition-based initialization. The new COFFO is prominent on CEC1 and CEC2 test instances in comparison to other algorithms.

When comparing various algorithms, contemporary computer science theory requires a statistical validation of the significance of improvements. The Friedman test [56, 57], a two-way variance analysis by ranks, demonstrates the considerable distinction between the proposed and other tested methods. Table 4 displays the ranking of twelve algorithms applied on ten functions.

**Table 4:** Friedman ranks of tested methods on CEC2019 benchmark functions.

Function	EHOI	EHO	SCA	SSA	GOA	WOA	BBO	MFO	PSO	COPSO	FFO	COFFO
CEC1	3	4	7	5	9	8	10	6	12	11	2	1
CEC2	3	4	9	5.5	7.5	5.5	10	7.5	12	11	2	1
CEC3	7.5	7.5	7.5	7.5	7.5	7.5	7.5	7.5	7.5	7.5	2	1
CEC4	3	4	12	6	10	11	8	9	7	5	2	1
CEC5	3	4	12	9	7.5	11	6	5	10	7.5	2	1
CEC6	8	10	12	3	6	9	5	4	11	7	1	2
CEC7	4	5	12	7	6	10	2	8	11	9	3	1
CEC8	3	4	12	6	10	11	5	9	7	8	2	1
CEC9	2	4	12	3	5	11	9	6	8	7	10	1
CEC10	1	12	10	5	3	6	9	4	7.5	7.5	11	2
Average	3.75	5.85	10.55	5.70	7.15	9.00	7.15	6.60	9.30	8.05	3.70	1.20
Rank	3	5	12	4	7	10	8	6	11	9	2	1

COFFO’s average ranking for the Friedman test is 1.20, thus demonstrating its superiority over the ten remaining algorithms (Table 4). At the significance level *α* = 0.005, the Friedman statistics ($\chi_{r}^{2}=60.2$) is greater than the *χ*^2^ critical value (*χ*^2^ = 19.7); hence the null hypothesis (*H*_0_) is rejected, allowing the conclusion that COFFO is substantially distinct from the rest of the compared methods.

Furthermore, Iman and Davenport’s test [58] is conducted since, as per [59], it can be more precise than the approximation of chi-square. The summary of the statistical results is given in Table 5.

**Table 5:** Resilts of Friedman and Iman-Davenport tests (*α* = 0.05).

Friedman value	*χ*^2^ critical value	*p*-value	Iman-Davenport value	*F* critical value	*p*-value
6.02 ⋅ 10^1^	1.97 ⋅ 10^1^	1.11 ⋅ 10^−16^	1.09 ⋅ 10^1^	1.89 ⋅ 10^0^	1.11 ⋅ 10^−13^

The *F*-distribution critical value (1.89) is less than the gained Iman-Davenport statistic of (10.9), so the second test rejects *H*_0_ as well. The significance level is greater than the *p*−*value* in both tests, as presented in Table 5.

Since both tests reject the null hypothesis, Holm’s step-down procedure, as a post-hoc procedure, is conducted with its results displayed in Table 6.

**Table 6:** Results of Holm’s step-down procedure.

Compared methods	p-value	Rank	0.05/(*k*−*i*)	0.1/(*k*−*i*)	*H*_01_	*H*_02_
COFFO vs SCA	3.34 ⋅ 10^−9^	0	0.004545	0.009091	1	1
COFFO vs PSO	2.54 ⋅ 10^−7^	1	0.005000	0.010000	1	1
COFFO vs WOA	6.58 ⋅ 10^−7^	2	0.005556	0.011111	1	1
COFFO vs COPSO	1.08 ⋅ 10^−5^	3	0.006250	0.012500	1	1
COFFO vs GOA	1.12 ⋅ 10^−4^	4	0.007143	0.014286	1	1
COFFO vs BBO	1.12 ⋅ 10^−4^	5	0.008333	0.016667	1	1
COFFO vs MFO	4.06 ⋅ 10^−4^	6	0.010000	0.020000	1	1
COFFO vs EHO	1.51 ⋅ 10^−3^	7	0.012500	0.025000	1	1
COFFO vs SSA	2.63 ⋅ 10^−3^	8	0.016667	0.033333	1	1
COFFO vs EHOI	5.69 ⋅ 10^−2^	9	0.025000	0.050000	0	0
COFFO vs FFO	6.05 ⋅ 10^−2^	10	0.050000	0.100000	0	1

The presented algorithm substantially surpassed ten out of eleven compared methods at significance level *α* = 0.1, with nine out of eleven at significance level *α* = 0.05.

In addition, a quad test [60] for the average fitness function is conducted, and the obtained *F* value is 8.53, while the *p*−*value* is 2.06*E*−10.

It can be concluded that the COFFO algorithm enhance the performance of the original FFO metaheuristic, thus affirming the goal of proposing an improved FFO algorithm.

**Fig 1:**
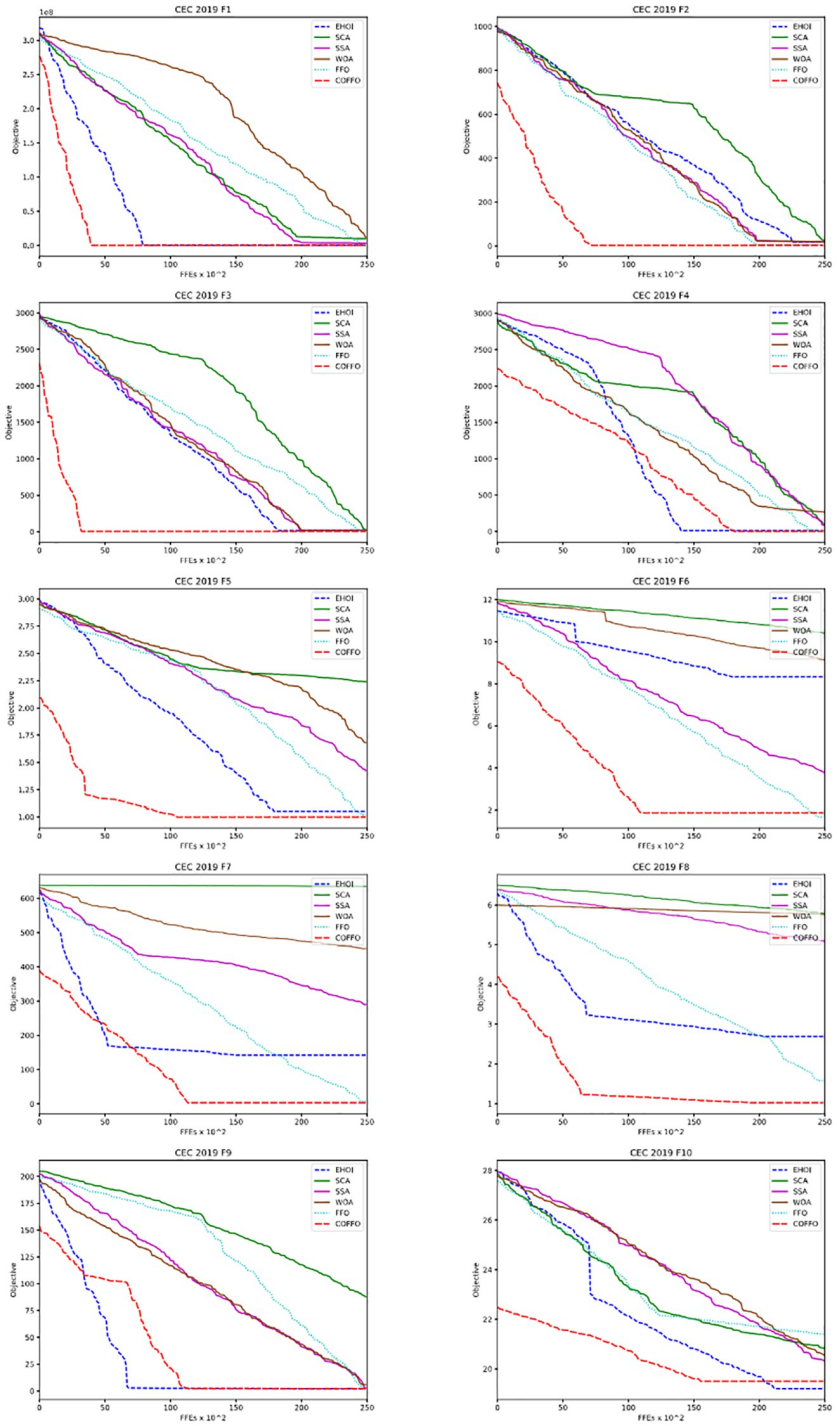
Convergence graphs for ten CEC 2019 benchmark functions and direct comparison between COFFO and FFO.

Next, Fig 1 displays convergence speed graphs for some algorithms. The best three, COFFO, FFO and EHOI, are emphasized in these graphs with different line styles. These show that the proposed COFFO algorithm has “starting adventage”, since its initialization utilizes chaotic sequences and opposition-based learning. Meaning, it has a better initial population then other algorithms, making the search easier.

## 5 Feature selection simulation results

The presented algorithm is substantiated on 21 standard datasets, collected from the UCI repository [61] and Arizona State University [62]. These feature selection datasets include: colon, arrhythmia, primary tumor, ILPD, ionosphere, leukemia, dermatology, zoo, glass, SCADI, SPECT heart, horse colic, libras movement, lung discrete, musk1, TOX 171, soybean, seeds, lymphography, LSVT and hepatitis. Details regarding datasets (number of features and training samples, dimensions)can be retrieved from [37]. Utilized datasets are devised of a diverse number of dimensions and features, of which leukemia and TOX 171 have the highest dimensionality, with 7070 and 5748 features respectively. Therefore, the performance of the presented algorithm is evaluated on disparate constructions that illustrate its effectiveness in divergent dimensions [63].

Five evaluation measures are determined to assess the performance of the algorithm. These measures represent the following: the best fitness value, the standard deviation of fitness value, the mean fitness value, feature selection ratio and classification accuracy.

### 5.1 Fitness evaluation and experimental conditions

The purpose of the fitness function is to estimate the quality of the solutions. Iteratively, every fruit fly is assessed by applying a fitness function. The fitness function in this research is selected to maximize classification accuracy and minimize the number of selected features. The fitness function is as follows [37]:

$$\begin{array}{c} Fit=\alpha ER+\beta \frac{\left|S\right|}{\left|O\right|} \end{array}$$ (9)

where *ER* is the classification’s error, |*S*| is the length of the subset of selected features, and |*O*| is the length of original features. Two weight infectors, *α* ∈ [0, 1] and *β* = (1−*α*), are used to indicate the influence of classification error and feature size on the fitness function. In COFFO, the transfer function is utilized to adapt the algorithm for binary problems. S-shaped and V-shaped transfer functions, named after the shape of the TF curve, are tested. V-shaped TF provided the best results and therefore implemented in the presented method. Table 7 provides the mathematical formulation of V-shaped transfer functions.

**Table 7:** V-shaped transfer functions.

Name	Transfer function
*V*_1_	$T\left(x\right)=|\text{erf}(\frac{\sqrt{\pi }}{2}x)|=|\frac{\sqrt{2}}{\pi }\int_{0}^{\frac{\sqrt{\pi }}{2}x}e^{-t^{2}}dt|$
*V*_2_	*T*(*x*) = |tanh(*x*)|
*V*_3_	$T\left(x\right)=|\frac{x}{\sqrt{1+x^{2}}}|$
*V*_4_	$T\left(x\right)=|\frac{2}{\pi }\text{arctan}(\frac{\pi }{2}x)|$

Modelled on the paper [37], the dataset is divided into the training and evaluation set utilising the stratified 10-fold cross-validation method. For wrapper-based FS, the K nearest neighbour (KNN, *k* = 5) is used to calculate classification error. The benefits of using KNN as a learning algorithm are its simplicity and low computational cost. All methods are conducted in 20 independent runs due to the non-deterministic nature of optimization algorithms. The averages of results are collected. The maximum number of iterations *maxIter* = 100 can produce a biased comparative analysis since number of utilized *FFEs* per iteration can vary between algorithms. Thus, the termination condition *maxFFes* = *N*+ N*maxIter is set to 1010. The population size is *N* = 10.

### 5.2 Comparison with other feature selection methods

This subsection provides the comparative performance analysis between the presented algorithm and eleven eminent algorithms: HLBDA, binary dragonfly algorithm (BDA) [35], binary multiverse optimizer (BMVO) [64], binary artificial bee colony (BABC) [65], binary particle swarm optimization (BPSO) [34], success-history based adaptive differential evolution with linear population size reduction (LSHADE) [66], chaotic crow search algorithm (CCSA) [45], evolution strategy with covariance matrix adaptation (CMAES) [67], binary coyote optimization algorithm (BCOA) [68, 69], COPSO and FFO. Table 8 shows the parameters for the compared algorithms.

**Table 8:** Control parameter settings for comparative feature selection methods.

Method	Parameter	Value
HLBDA	pl	0.4
	gl	0.7
BDA	All controlling parameters	Identical to the original paper
BABC	Maximum limits	5
BMVO	WEP	[0.02, 1]
	TDR	[0.6, 0]
BPSO	Inertia weight, w	[0.9, 0.4]
	Acceleration factors, c1 and c2	2
CCSA	AP	0.1
	fl	2
BCOA	Coyote number	5
	Paks number	2
CMAES	Parents number	λ/4
LSHADE	Minimum size of population	4
	Size of memory	5

The personal learning rate (pl) and global learning rate (gl) of HLBDA are set to 0.4 and 0.7, respectively. The maximum limit for BABC is set at 5. The wormhole existence probability (WEP) increases from 0.02 to 1 whilst the traveling distance rate (TDR) decreases from 0.6 to 0—both in BMVO. In BPSO, acceleration factors are set at 2 and the inertia weight is decreasing from 0.9 to 0.4. In CCSA the awareness probability (AP) and flight length (fl) are set at 0.1 and 2, respectively. In BCOA, the number of coyotes and packs are set to 5 and 2. The number of parents for CMAES is set at 25% of solutions. When it comes to LSHADE, the memory size and minimum population size are set at 5 and 4.

Tables 9–11 show testing results of mean fitness, the best fitness, and the standard deviation of fitness function for the presented COFFO. As shown in Table 9, COFFO identified the optimal best fitness value on fifteen datasets, accompanied by HLBDA in eight datasets.

**Table 9:** Best fitness value results for tested algorithms.

No.	Dataset	Best fitness value											
		HLBDA	BDA	BABC	BMVO	BPSO	CCSA	BCOA	CMAES	LSHADE	COPSO	FFO	COFFO
1	Glass	**0.0067**	**0.0067**	**0.0067**	**0.0067**	**0.0067**	**0.0067**	**0.0067**	**0.0067**	**0.0067**	**0.0067**	**0.0067**	**0.0067**
2	Hepatitis	**0.1154**	0.1244	0.1304	0.1224	0.1237	0.1310	0.1219	0.1231	0.1234	0.1231	0.1211	0.1199
3	Lymphography	0.1117	0.1180	0.1121	0.1295	0.1178	0.1310	0.1255	0.1170	0.1224	0.1186	0.1176	**0.1115**
4	Primary Tumor	0.5647	0.5730	0.5675	0.5888	0.5623	0.5755	0.5642	0.5623	0.5880	0.5763	0.5729	**0.5620**
5	Soybean	0.2010	0.2073	0.2037	0.2420	0.2190	0.2293	0.2037	0.2010	0.2038	0.2037	0.2069	**0.2008**
6	Horse Colic	0.1300	0.1329	0.1349	0.1439	0.1311	0.1418	0.1303	0.1300	0.1327	0.1336	0.1327	**0.1295**
7	Ionosphere	**0.0695**	0.0730	0.0831	0.0980	0.0816	0.0904	0.0715	0.0745	0.0720	0.0749	0.0752	0.0718
8	Zoo	0.0332	0.0325	0.0332	0.0332	0.0325	0.0337	0.0325	0.0333	0.0325	0.0332	0.0332	**0.0323**
9	Musk 1	**0.0608**	0.0625	0.0880	0.0940	0.0782	0.0834	0.0663	0.0740	0.0633	0.0633	0.0638	0.6039
10	Arrhythmia	0.2927	0.3180	0.3329	0.3351	0.3280	0.3400	0.3105	0.3271	0.3000	0.2991	0.3086	**0.2922**
11	Dermatology	0.0130	0.0133	0.0161	0.0216	0.0158	0.0184	0.0160	0.0134	0.0160	0.0158	0.0134	**0.0128**
12	SPECT Heart	0.1385	0.1385	0.1413	0.1455	0.1359	0.1409	0.1336	0.1388	0.1398	0.1385	0.1359	**0.1333**
13	Libras Movement	0.1667	0.1810	0.1940	0.2020	0.1912	0.1915	0.1720	0.1749	0.1690	0.1702	0.1795	**0.1662**
14	ILPD	**0.2672**	**0.2672**	0.2721	0.2699	**0.2672**	**0.2672**	**0.2672**	**0.2672**	**0.2672**	**0.2672**	0.2678	0.2678
15	Seeds	**0.0453**	**0.0453**	**0.0453**	**0.0453**	**0.0453**	**0.0453**	**0.0453**	**0.0453**	**0.0453**	**0.0453**	**0.0453**	**0.0453**
16	LSVT	**0.2389**	0.2695	0.3001	0.2797	0.2619	0.2980	0.3007	0.2865	0.3009	0.2834	0.2713	0.2596
17	SCADI	0.1158	0.1168	0.1310	0.1311	0.1176	0.1319	0.1159	0.1165	0.1162	0.1159	0.1159	**0.1151**
18	TOX 171	0.1260	0.1378	0.1898	0.1958	0.1720	0.1735	0.1484	0.1960	0.1383	0.1377	0.1424	**0.1258**
19	Leukemia	0.0311	0.0313	0.0458	0.0444	0.0456	0.0580	0.0308	0.0456	0.0313	0.0311	0.0314	**0.0308**
20	Lung discrete	0.0554	0.0599	0.0830	0.0845	0.0717	0.0829	0.0658	0.0736	0.0559	0.0584	0.0602	**0.0551**
21	Colon	**0.0823**	0.0966	0.1152	0.1157	0.1130	0.1300	0.0987	0.1134	0.0994	0.0986	0.1018	0.0892

Results in Table 10 display that COFFO detected the optimal mean fitness value in fourteen datasets, followed by HLBDA with four. These results entail that the presented COFFO can locate the optimal feature subset in most cases, yielding a satisfying performance.

**Table 10:** Mean fitness value results for tested algorithms.

No.	Dataset	Mean fitness value											
		HLBDA	BDA	BABC	BMVO	BPSO	CCSA	BCOA	CMAES	LSHADE	COPSO	FFO	COFFO
1	Glass	0.0112	0.0112	**0.0111**	0.0116	**0.0111**	0.0116	**0.0111**	0.0118	0.0116	0.0111	0.0114	0.0113
2	Hepatitis	**0.1312**	0.1369	0.1384	0.1452	0.1335	0.1455	0.1424	0.1428	0.1400	0.1325	0.1402	0.1337
3	Lymphography	0.1312	0.1360	0.1351	0.1525	0.1340	0.1513	0.1418	0.1394	0.1475	0.1328	0.1353	**0.1306**
4	Primary Tumor	0.5849	0.5933	0.5845	0.6086	0.5849	0.5998	0.5943	0.5935	0.5990	0.5845	0.5898	**0.5839**
5	Soybean	0.2125	0.2214	0.2256	0.2593	0.2246	0.2481	0.2170	0.2179	0.2209	0.2218	0.2206	**0.2119**
6	Horse Colic	0.1360	0.1429	0.1481	0.1673	0.1410	0.1701	0.1432	0.1420	0.1481	0.1392	0.1413	**0.1353**
7	Ionosphere	**0.0843**	0.0930	0.1014	0.1107	0.0960	0.1112	0.0871	0.0883	0.0911	0.0947	0.0976	0.0929
8	Zoo	0.0400	0.0408	0.0400	0.0480	**0.0368**	0.0494	0.0439	0.0471	0.0501	0.0369	0.0434	0.0382
9	Musk 1	0.0674	0.0832	0.0959	0.1081	0.0930	0.1018	0.0791	0.0844	0.0795	0.0911	0.0819	**0.0668**
10	Arrhythmia	0.3159	0.3340	0.3451	0.3540	0.3415	0.3528	0.3292	0.3351	0.3272	0.3304	0.3286	**0.3147**
11	Dermatology	0.0173	0.0192	0.0202	0.0254	0.0195	0.0238	0.0209	0.0181	0.0198	0.0196	0.0197	**0.0166**
12	SPECT Heart	0.1507	0.1543	0.1573	0.1705	0.1541	0.1632	0.1612	0.1646	0.1600	0.1540	0.1545	**0.1503**
13	Libras Movement	0.1814	0.1938	0.2026	0.2094	0.2005	0.2074	0.1858	0.1889	0.1900	0.1952	0.1927	**0.1809**
14	ILPD	0.2789	0.2788	0.2803	0.2815	0.2792	0.2801	0.2815	0.2844	0.2835	0.2793	0.2801	**0.2782**
15	Seeds	0.0557	0.0557	**0.0529**	0.0533	**0.0529**	**0.0529**	0.0532	0.0538	0.0559	**0.0529**	0.0537	0.0530
16	LSVT	**0.3008**	0.3165	0.3173	0.3169	0.3174	0.3294	0.3280	0.3204	0.3337	0.3171	0.3201	0.3082
17	SCADI	0.1260	0.1311	0.1344	0.1415	0.1331	0.1412	0.1292	0.1285	0.1264	0.1329	0.1321	**0.1255**
18	TOX 171	0.1577	0.1836	0.2138	0.2371	0.2072	0.2299	0.1816	0.2197	0.1778	0.1992	0.1829	**0.1572**
19	Leukemia	0.0509	0.0625	0.0696	0.0754	0.0609	0.0741	0.0553	0.0681	0.0628	0.0591	0.0616	**0.0501**
20	Lung discrete	0.0774	0.0834	0.0940	0.1041	0.0908	0.0981	0.0832	0.0892	0.0811	0.0867	0.0848	**0.0765**
21	Colon	**0.1331**	0.1500	0.1628	0.1701	0.1506	0.1684	0.1434	0.1603	0.1436	0.1505	0.1548	0.1436

As shown in Table 11, COFFO discerned the lowest standard deviation in twelve datasets, accompanied by BABC with four. COFFO consistently obtained better results compared to FFO.

**Table 11:** Standard deviation of fitness value results for tested algorithms.

No.	Dataset	Standard deviation of fitness value											
		HLBDA	BDA	BABC	BMVO	BPSO	CCSA	BCOA	CMAES	LSHADE	COPSO	FFO	COFFO
1	Glass	0.0033	0.0033	0.0033	0.0033	0.0033	**0.0032**	0.0033	0.0036	0.0033	0.0033	0.0033	0.0033
2	Hepatitis	0.0094	0.0063	0.0059	0.0100	0.0079	0.0065	0.0138	0.0133	0.0092	0.0070	0.0068	**0.0057**
3	Lymphography	0.0129	0.0120	0.0126	0.0105	0.0140	0.0115	0.0133	0.0151	0.0148	0.0134	0.0122	**0.0101**
4	Primary Tumor	0.0103	0.0100	0.0086	0.0081	0.0114	0.0118	0.0136	0.0136	0.0135	0.0118	0.0102	**0.0080**
5	Soybean	0.0088	0.0095	0.0088	0.0120	**0.0043**	0.0110	0.0102	0.0114	0.0111	0.0065	0.0099	0.0061
6	Horse Colic	**0.0040**	0.0090	0.0075	0.0163	0.0071	0.0141	0.0109	0.0099	0.0156	0.0078	0.1008	0.0079
7	Ionosphere	0.0086	0.0104	0.0102	0.0054	0.0056	0.0091	0.0105	0.0071	0.0100	0.0059	0.0092	**0.0053**
8	Zoo	0.0080	0.0081	0.0072	0.0101	0.0067	0.0098	0.0093	0.0100	0.0114	0.0071	0.0082	**0.0063**
9	Musk 1	**0.0063**	0.0100	0.0064	0.0075	0.0079	0.0079	0.0078	0.0075	0.0098	0.0075	0.0081	0.0075
10	Arrhythmia	0.0094	0.0089	**0.0044**	0.0075	0.0073	0.0066	0.0099	0.0059	0.0131	0.0069	0.0090	0.0069
11	Dermatology	0.0020	0.0034	0.0023	0.0025	0.0020	0.0039	0.0025	0.0023	0.0035	0.0023	0.0027	**0.0019**
12	SPECT Heart	**0.0068**	0.0083	0.0082	0.0096	0.0078	0.0097	0.0203	0.0185	0.0169	0.0079	0.0090	0.0078
13	Libras Movement	0.0084	0.0103	0.0086	0.0051	0.0075	0.0091	0.0101	0.0089	0.0122	0.0073	0.0078	**0.0049**
14	ILPD	0.0050	0.0049	0.0049	0.0054	0.0047	0.0051	0.0063	0.0081	0.0065	0.0049	0.0050	**0.0045**
15	Seeds	0.0152	0.0152	**0.0057**	0.0060	**0.0057**	**0.0057**	0.0061	0.0066	0.0151	**0.0057**	0.0061	0.0059
16	LSVT	0.0312	0.0273	0.0190	0.0209	0.0243	0.0221	0.0227	0.0201	0.0217	0.0204	0.0203	**0.0186**
17	SCADI	0.0081	0.0091	0.0058	0.0064	0.0063	0.0066	0.0117	0.0074	0.0080	0.0060	0.0076	**0.0056**
18	TOX 171	0.0213	0.0273	0.0179	0.0156	0.0177	0.0200	0.0181	0.0141	0.0213	0.0176	0.0182	**0.0138**
19	Leukemia	0.0134	0.0153	0.0137	0.0156	0.0114	0.0142	0.0134	0.0113	0.0199	0.0113	0.0136	**0.0111**
20	Lung discrete	0.0095	0.0105	**0.0073**	0.0092	0.0090	0.0085	0.0110	0.0078	0.0102	0.0985	0.0094	0.0081
21	Colon	0.0335	0.0350	**0.0257**	0.0305	0.0274	0.0264	0.0296	0.0335	0.0269	0.0273	0.0308	0.0273

**Fig 2:**
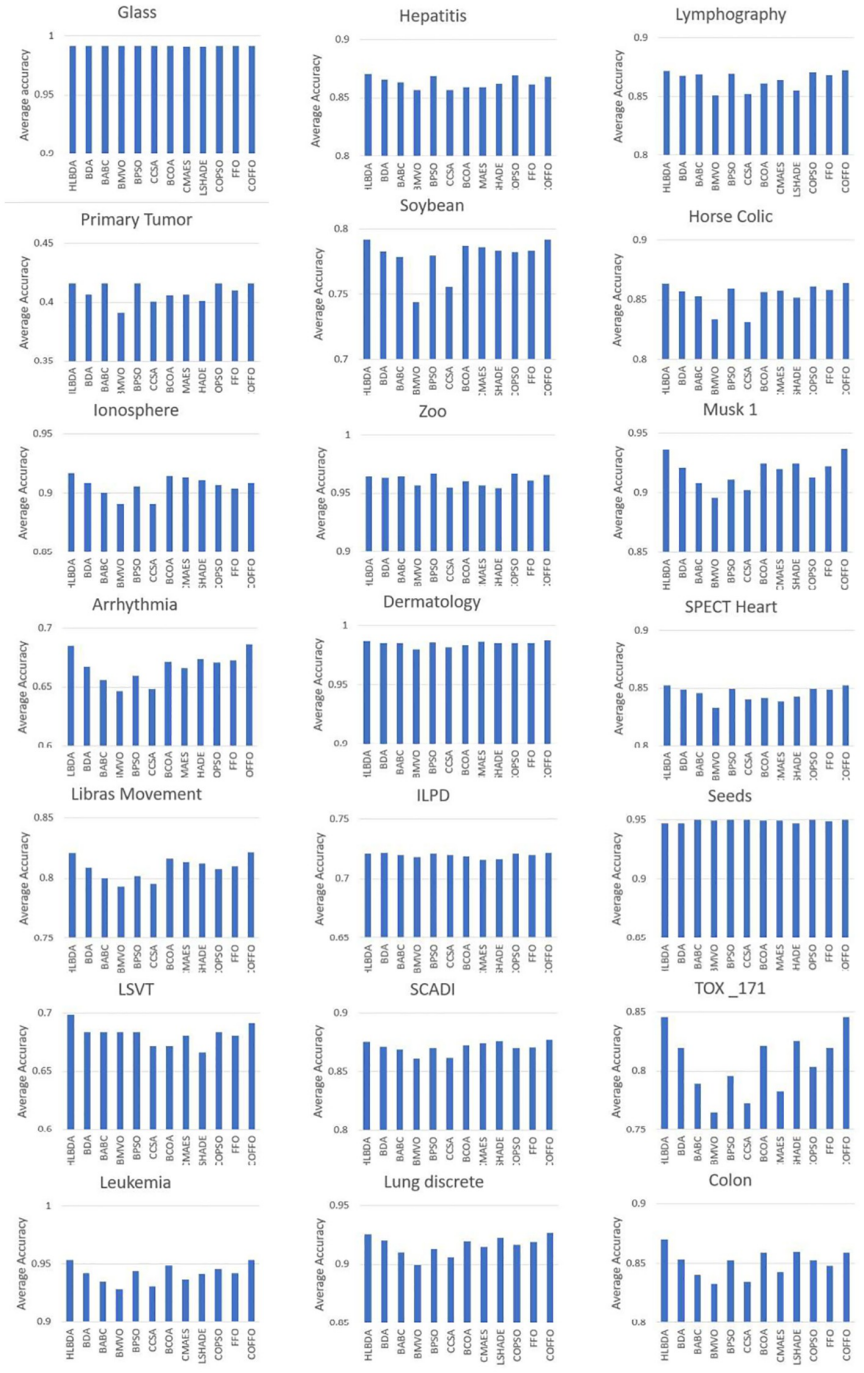
The result of the accuracy of tested algorithms.

Fig 2 provides the classification accuracy result of tested algorithms. As demonstrated, COFFO obtained the highest accuracy in 12 datasets, exceeding the remaining algorithms in procuring the optimal feature subset.

Boxplot is a type of chart often used in explanatory data analysis. It shows minimum score (the lowest score, excluding outliers), lower quartile (25% of scores fall below the lower quartile value), median (marks the mid-point of the data), upper quartile (75% of the scores fall below the upper quartile value), maximum score (the highest score, excluding outliers), whiskers (represent scores outside the middle 50%) and the interquartile range (IQR) (box plot displaying the middle 50% of scores). The average error rate was taken for all 21 datasets from which the boxplots analysis is conducted, to exhibit the stability, i.e. diversification of the proposed algorithm.

**Fig 3:**
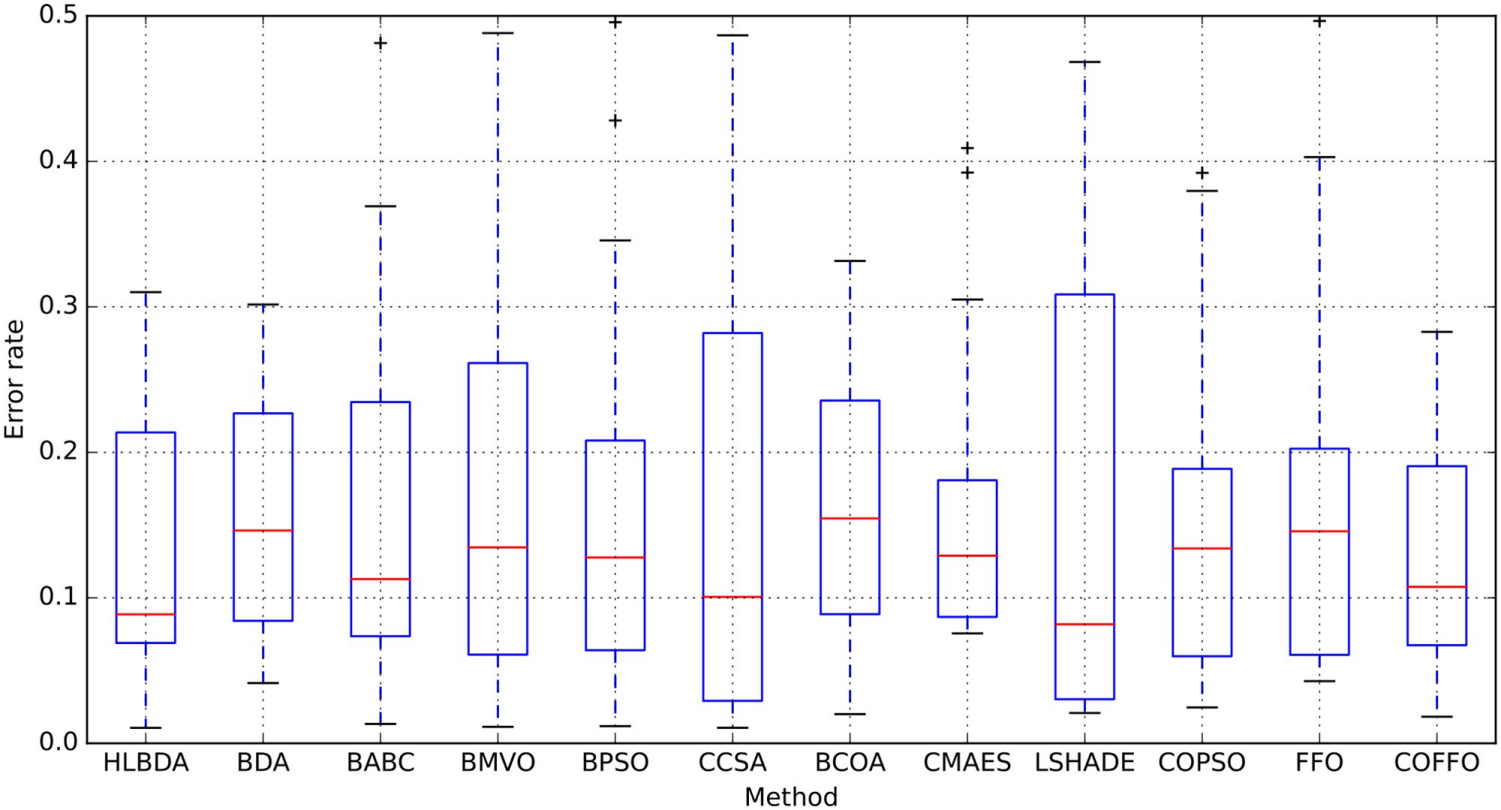
Boxplots analysis of the tested algorithms using average error rate across 21 datasets.

Fig 3 provides the boxplots analysis of eleven different algorithms. As seen in Fig 3, the presented COFFO is relatively stable, and in comparison to second best HLBDO and original FFO, has smaller IQR, that is, lower dispersion and the best maximal score. The gained results uphold the effectiveness of the proposed algorithm in maintaining the highest classification accuracy.

Table 12 shows the feature selection ratio results. The length of the optimal feature subset obtained by algorithms is proportional to the feature selection ratio—the smaller the subset is, the lower the ratio. The results display that COFFO attained the smallest feature size in thirteen datasets, accompanied by HLBDA with seven. Compared to other algorithms, COFFO can frequently find a small, most informative subset of features. Indisputable, COFFO is efficient in selecting the best feature selection solution and preventing the local optima.

**Table 12:** Feature selection ratio results for tested algorithms.

No.	Dataset	Feature selection ratio											
		HLBDA	BDA	BABC	BMVO	BPSO	CCSA	BCOA	CMAES	LSHADE	COPSO	FFO	COFFO
1	Glass	**0.2900**	**0.2900**	0.3050	0.3250	0.3050	0.3300	0.3050	**0.2900**	0.3050	0.3050	**0.2900**	**0.2900**
2	Hepatitis	0.3185	0.3660	0.3159	0.3604	0.3261	0.3527	0.3053	0.3501	0.3370	0.3254	0.3248	**0.3046**
3	Lymphography	0.4499	0.4806	0.5082	0.4890	0.5002	0.4973	0.4471	0.5085	0.4334	0.4811	0.4652	**0.4319**
4	Primary Tumor	0.6678	0.6120	0.6707	0.5943	0.6675	0.6439	0.6060	0.6264	0.6414	0.6618	0.6071	**0.5932**
5	Soybean	0.6530	0.6230	0.6429	0.5745	0.6415	0.5972	0.6373	0.6284	0.6485	0.6109	0.6091	**0.5734**
6	Horse Colic	**0.0871**	0.1368	0.2425	0.2576	0.1964	0.2926	0.1055	0.1129	0.1501	0.1746	0.1218	0.1023
7	Ionosphere	0.2191	0.2678	0.2899	0.2881	0.2439	0.3425	0.2266	0.2455	0.2822	0.2437	0.2476	**0.2177**
8	Zoo	0.4562	0.4469	0.4969	0.5189	0.4251	0.4937	0.4532	0.4499	0.4938	0.4235	0.4463	**0.4242**
9	Musk 1	0.4686	0.4782	0.4946	0.4604	0.4963	0.5033	0.4460	0.4947	0.4848	0.4952	0.4607	**0.4453**
10	Arrhythmia	0.4051	0.4699	0.4805	0.4302	0.4706	0.4789	**0.4050**	0.4628	0.4496	0.4613	0.4381	0.4098
11	Dermatology	0.4575	0.4826	0.5572	0.5425	0.5341	0.5425	0.4370	0.4926	0.5074	0.5087	0.4819	**0.4365**
12	SPECT Heart	0.4477	0.4499	0.4866	0.5274	0.5044	0.5249	**0.4161**	0.4751	0.4274	0.5038	0.4614	0.4386
13	Libras Movement	0.4161	0.4518	0.4598	0.4313	0.4488	0.4645	0.4061	0.4302	0.4323	0.4486	0.4256	**0.4043**
14	ILPD	0.2950	0.3150	0.3350	**0.2800**	0.3250	0.3200	0.3050	0.3450	0.2950	0.3150	0.2950	**0.2800**
15	Seeds	**0.3143**	**0.3143**	**0.3143**	0.3214	**0.3143**	**0.3143**	0.3214	0.3430	0.3214	0.3143	0.3214	0.3214
16	LSVT	**0.2845**	0.3483	0.4501	0.4007	0.4426	0.4495	0.2986	0.4199	0.3166	0.4297	0.3781	0.3127
17	SCADI	**0.2888**	0.3728	0.4372	0.4154	0.4250	0.4523	0.3182	0.3980	0.3487	0.4259	0.3972	0.3736
18	TOX 171	0.4796	0.4831	0.5001	0.4453	0.4974	0.4984	0.4592	0.4981	0.4960	0.4982	0.4597	**0.4445**
19	Leukemia	0.4573	0.4696	0.4910	0.4177	0.4945	0.4934	0.4146	0.4913	0.4776	0.4910	0.4268	**0.4140**
20	Lung discrete	**0.3713**	**0.4391**	0.4862	0.4482	0.4809	0.4855	0.3808	0.4704	0.4378	0.4518	0.4467	0.3909
21	Colon	**0.4380**	0.4630	0.4865	0.4442	0.4909	0.4886	0.4179	0.4884	0.4667	0.4880	0.4522	0.4391

For the statistical analysis, the Wilcoxon signed-rank test [70] is conducted for COFFO comparison against other methods. If the *p*−*value* is smaller than 0.05, then the classification accuracy of the two compared methods is significantly different. Table 13 displays the results of the Wilcoxon test of COFFO as opposed to other methods. The results acquired prove that COFFO’s classification performance is significantly better than the remaining candidates in all cases except for HLBDA.

**Table 13:** The result of the Wilcoxon test of presented COFFO against compared methods.

	HLBDA	BDA	BABC	BMVO	BPSO	CCSA	BCOA	CMAES	LSHADE	COPSO	FFO
p-value	1.01 ⋅ 10^−1^	9.54 ⋅ 10^−7^	2.38 ⋅ 10^−6^	4.77 ⋅ 10^−7^	3.34 ⋅ 10^−5^	9.54 ⋅ 10^−7^	6.53 ⋅ 10^−5^	4.77 ⋅ 10^−6^	6.13 ⋅ 10^−5^	5.25 ⋅ 10^−5^	4.77 ⋅ 10^−7^

Particular emphasis should be placed on COFFO’s performance in high-dimensional datasets, such as *TOX*171 and *Leukemia*. Experimental results indicate that the proposed approach is more effective in selecting relevant features than the original FFO and other tested methods.

**Fig 4:**
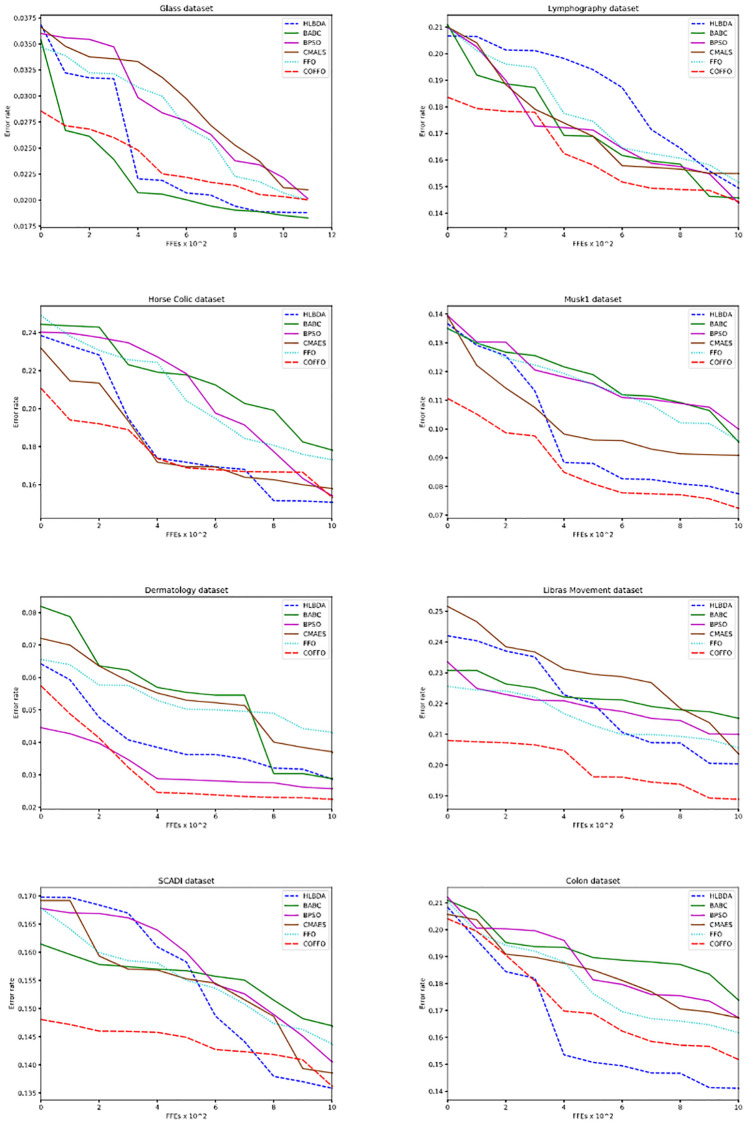
Error rate convergence graphs.

For extensive analysis, error rate convergence graphs of COFFO, FFO and six more methods on eight datasets are provided in Fig 4.

The introduced COFFO generated the best initial population on six out of eight datasets, thus showing a considerable advantage of chaotic-based and opposition-based learning implementation. BPSO obtained the best results in its initial phase on a *Dermatology* dataset, while all tested algorithms gave a similar performance on the *Colon* dataset. The proposed COFFO is drastically better when generating the initial population than the original FFO in most datasets.

## 6 COVID-19 dataset and results

What started as an acute respiratory syndrome outbreak in China quickly became a pandemic. The SARS-CoV-2, also called COVID-19, has caused the deaths of millions of people worldwide since its beginning [71, 72]. Artificial intelligence can help with the prevention, detection and diagnosis of COVID-19 [73]. This section displays the implementation of the proposed algorithm in COVID-19 patient health prediction. The dataset of COVID-19 cases was gathered from the [74]. Table 14 shows fifteen features contained within the said dataset. The aim is to predict the death and recovery conditions determined by specific factors. Solely the data containing values for “death” and “recov” status are considered. For validation, the data is divided equally into two disjunct sets—training and testing. Each feature has a numeric form assigned to it.

**Table 14:** COVID-19 dataset description.

No.	Feature	Description
1	id	The ID of patients
2	location	Patients’ location
3	country	Patients’ country
4	gender	Patients’ gender
5	age	Patients’ age
6	sym on	Patients’ symptoms date
7	hosp vis	Patients’ hospital visit date
8	vis wuhan	Previous patient visit to Wuhan, China
9	from wuhan	Patient is a resident of Wuhan, China
10	symptom 1	Clinical symptom
11	symptom 2	Clinical symptom
12	symptom 3	Clinical symptom
13	symptom 4	Clinical symptom
14	symptom 5	Clinical symptom
15	symptom 6	Clinical symptom

As Table 15 shows the proposed COFFO has optimal mean fitness value, best fitness value and feature selection ratio value, followed by HLBDA.

**Table 15:** The result of the best fitness value, mean fitness value, standard deviation of fitness value and feature selection ratio of algorithms on the COVID-19 dataset.

	HLBDA	BDA	BABC	BMVO	BPSO	CCSA	BCOA	CMAES	LSHADE	COPSO	FFO	COFFO
Best fitness	0.0679	0.0683	0.0701	0.0718	0.0712	0.0715	0.0697	0.0706	0.0713	0.0706	0.0701	**0.0676**
Mean fitness	0.0778	0.0793	0.0804	0.0825	0.0824	0.0839	0.0786	0.0809	0.0812	0.0804	0.0800	**0.0761**
Standard deviation	0.0082	0.0087	0.0075	**0.0073**	0.0091	0.0094	0.0118	0.0121	0.0108	0.0089	0.0089	0.0084
Feature selection ratio	0.1627	0.1740	0.2101	0.2427	0.1540	0.2313	0.1593	0.1987	0.1807	0.1536	0.1753	**0.1527**

**Fig 5:**
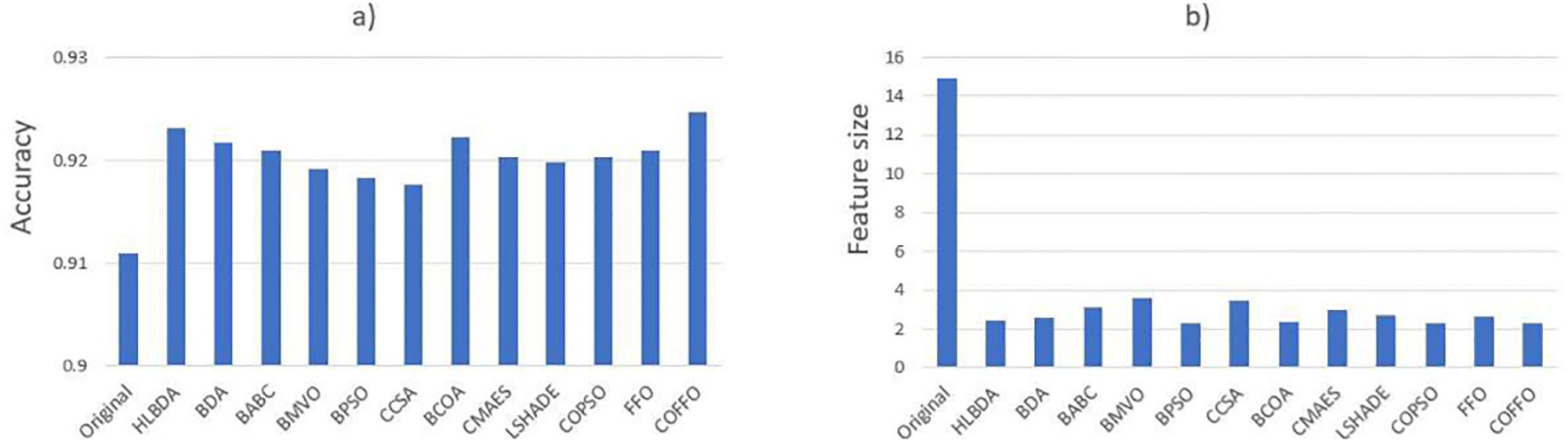
a) Accuracy and b) selected feature size of algorithms on the COVID-19 dataset.

Fig 5 demonstrates the average accuracy and selected feature size of all compared algorithms tested on the COVID-19 dataset. COFFO outperformed the original FFO and other algorithms by attaining the average classification accuracy of 92.46% and the smallest feature size of 2.29. According to the collected data, the most selected features were *gender*, *age* and *symptom*2. On the other hand, *id* and *symptom*6 were never selected by COFFO algorithm. The results indicate that these features are ineffective in discerning the data patterns in patient health prediction procedure. The accuracy of patient health prediction can be more precise in the future by gathering additional clinical features.

## 7 Discussion

The results illustrate that COFFO has shown the best performance in selecting relevant features while significantly reducing dimensionality.

The improvement is reflected in both exploration and exploitation stages. Due to the fixed position update strategy, the original FFO can get stuck in the local optima in its exploration phase. To solve this problem, opposition-based learning and mapping solutions to generating chaotic sequences have been implemented, thus achieving an initial population that is closer to an optimum region of the search space and accelerating convergence towards the optimal global solution in a complex feature space. Further, the exploitation phase has been improved with a chaotic local search strategy for fine-tuned exploitation. The disadvantage of implementing chaotic opposition-based learning in the initial phase, and chaotic local search in the exploitation phase, is the increase in time complexity.

## 8 Conclusion

This study presents a novel chaotic oppositional fruit fly optimization algorithm (COFFO), a wrapper-based technique for feature selection. The COFFO employs chaotic-based and opposition-based learning to improve the performance of the original algorithm. With the current praxis in mind regarding the optimization process, the introduced algorithm is tested on ten unconstrained benchmark functions from CEC2019. For comparative analysis, eleven other well-known metaheuristic methods are tested under the same experimental conditions. The mean fitness and standard deviation are compared between tested algorithms, and, additionally, statistical tests are conducted, which prove that COFFO outperforms all the other tested methods. Further, the proposed approach outperforms the original FFO significantly.

The next phase centres on applying COFFO to 21 standard datasets. For performance comparison, eleven other well-known approaches are tested under the same experimental conditions. The best fitness value, the mean fitness value, standard deviation, accuracy and feature selection ratio are used for comparison. Wilcoxon statistical test is conducted, as well, for testing the proposed COFFO against other methods. In all the above-noted datasets, COFFO outscored tested algorithms in most cases, specifically on high-dimensional feature sets.

Finally, COFFO is employed in COVID-19 patient health prediction, where the introduced algorithm achieved excellent performance surpassing preceding algorithms. Among the peers, especially as opposed to the original FFO, COFFO can select a subset of significant features with high discriminatory capacities. Taking everything into account, the presented COFFO not only obtains the highest classification accuracy but is also effective in dimensionality reduction.

As part of the future research proposed COFFO can be tested on various NP-hard optimization challenges from domains such as cloud computing, wireless sensor networks, portfolio optimization and also applied for enhancing machine learning models.

## Supporting information

S1 Dataset (ZIP)
